# Knockdown of *TFRC* suppressed the progression of nasopharyngeal carcinoma by downregulating the PI3K/Akt/mTOR pathway

**DOI:** 10.1186/s12935-023-02995-7

**Published:** 2023-08-29

**Authors:** Guofei Feng, Yasushi Arima, Kaoru Midorikawa, Hatasu Kobayashi, Shinji Oikawa, Weilin Zhao, Zhe Zhang, Kazuhiko Takeuchi, Mariko Murata

**Affiliations:** 1 Department of Environmental and Molecular Medicine, Mie University Graduate School of Medicine, Tsu, 514-8507 Mie Japan; 2 Department of Otorhinolaryngology-Head and Neck Surgery, Mie University Graduate School of Medicine, Tsu, 514-8507 Mie Japan; 3 Graduate School of Health Science, Suzuka University of Medical Science, Suzuka, 510-0226 Mie Japan; 4 Department of Otorhinolaryngology-Head and Neck Surgery, First Affiliated Hospital of Guangxi Medical University, Nanning, 530021 Guangxi China

**Keywords:** Nasopharyngeal carcinoma, TFRC, PI3K/Akt/mTOR signaling pathway, RNA-seq, siRNA

## Abstract

**Background:**

The transferrin receptor (TfR) encoded by *TFRC* gene is the main cellular iron importer. TfR is highly expressed in many cancers and is expected to be a promising new target for cancer therapy; however, its role in nasopharyngeal carcinoma (NPC) remains unknown.

**Methods:**

The TfR levels were investigated in NPC tissues and cell lines using immunohistochemistry and reverse transcription-quantitative polymerase chain reaction. Knockdown of *TFRC* using two siRNA to investigate the effects on intracellular iron level and biological functions, including proliferation by CKK-8 assay, colony formation, cell apoptosis and cell cycle by flow cytometry, migration and invasion, and tumor growth in vivo by nude mouse xenografts. RNA sequencing was performed to find possible mechanism after *TFRC* knockdown on NPC cells and further verified by western blotting.

**Results:**

TfR was overexpressed in NPC cell lines and tissues. Knockdown of *TFRC* inhibited cell proliferation concomitant with increased apoptosis and cell cycle arrest, and it decreased intracellular iron, colony formation, migration, invasion, and epithelial-mesenchymal transition in HK1-EBV cells. Western blotting showed that *TFRC* knockdown suppressed the levels of the iron storage protein FTH1, anti-apoptotic marker BCL-xL, and epithelial-mesenchymal transition markers. We confirmed in vivo that *TFRC* knockdown also inhibited NPC tumor growth and decreased Ki67 expression in tumor tissues of nude mouse xenografts. RNA sequencing and western blotting revealed that *TFRC* silencing inhibited the PI3K/Akt/mTOR signaling pathway.

**Conclusions:**

These results indicated that TfR was overexpressed in NPC, and *TFRC* knockdown inhibited NPC progression by suppressing the PI3K/Akt/mTOR signaling pathway. Thus, TfR may serve as a novel biomarker and therapeutic target for NPC.

**Supplementary Information:**

The online version contains supplementary material available at 10.1186/s12935-023-02995-7.

## Background

Nasopharyngeal carcinoma (NPC) is a malignancy of the nasopharyngeal mucosal lining. NPC has an unbalanced geographical distribution, and more than 70% of new cases occur in East and Southeast Asia, especially in Guangdong and Guangxi, China. Owing to lifestyle and environmental changes, the incidence and mortality rates have gradually decreased over the past few decades [1]. However, because the absence of obvious clinical signs in the early stages makes diagnosis difficult, 70% of patients are diagnosed at advanced stages and have a poor prognosis [2, 3]. Therefore, there is an urgent need to clarify the biological mechanisms underlying NPC pathogenesis and identify novel biomarkers for the early clinical diagnosis and treatment of NPC.

Iron is an essential trace element involved in a variety of fundamental processes in humans, including DNA synthesis, ATP production, and oxygen transportation [4, 5]. Iron homeostasis is tightly regulated in normal cells. As free radicals can form during iron metabolism, disturbances in iron homeostasis can increase the risk of cancer and are associated with carcinogenesis [6]. Numerous studies have shown that the disruption of iron homeostasis is one of the metabolic hallmarks of malignant cancer cells and that iron is required for tumor development, survival, proliferation, and metastasis [7]. Many iron metabolism-related proteins and pathways are altered in malignancies, indicating the critical role of iron in cancer [8]. In addition, compared with normal cells, cancer cells are more dependent on iron for growth, making them more sensitive to iron deprivation; this is known as “iron addiction” [9, 10]. In our previous study, we also found that elevated iron levels can increase proliferation, migration, and invasion abilities and that iron deprivation can decrease proliferation in NPC cells, indicating the importance of iron in NPC [11]. The transferrin receptor (TfR), also known as CD71, is a vital cellular iron uptake receptor that can input extracellular iron by interacting with the extracellular iron (Fe^3+^)-bound transferrin complex [12]. Generally, TfR expression is low in most normal cells but high in erythroblasts and rapidly proliferating cells, such as cancer cells [13]. TfR overexpression has been found in many human tumors, such as liver, breast, lung, brain, thyroid, ovarian, colon, and esophageal cancer [14–21]. Moreover, interest continues to increase in both targeting TfR as a direct anticancer agent and its use for delivery purposes [12]. However, the expression patterns and molecular mechanisms of TfR in NPC have not yet been elucidated.

In this study, we explored the expression of TfR in NPC and investigated the biological function changes both in vivo and in vitro after knockdown of *TFRC*. We further explored the possible mechanism by RNA sequencing (RNA-seq) and verified it using western blotting. The results showed that TfR was upregulated in NPC and knockdown of *TFRC* suppressed cell proliferation, concomitant with increased apoptosis and cell cycle arrest in NPC cells. Intracellular iron level, migration, invasion, and epithelial–mesenchymal transition (EMT) were inhibited after knockdown of *TFRC*. We further confirmed that *TFRC* knockdown inhibited NPC progression via the PI3K/Akt/mTOR signaling pathway.

## Methods

### Bioinformatic analysis

The mRNA expression of *TFRC* in different cancers was analyzed using the Gene Expression Profiling Interactive Analysis (GEPIA) database [22], and survival analysis was used to determine the relationship between *TFRC* levels and overall survival time in patients with head and neck squamous cell carcinoma. Using the Gene Expression Omnibus (GEO) database 2R tools, the mRNA expression of *TFRC* in NPC was analyzed in four microarray datasets (GSE53819, GSE12452, GSE34573, and GSE13597) containing NPC and normal control tissues.

### Human tissues

An NPC tissue microarray (HNasC070PG01) was purchased from Shanghai Outdo Biotech Co., Ltd. that contained 70 NPC tissue samples. Fifteen normal nasopharyngeal epithelial (NNE) samples from patients without cancer were obtained as controls from the Department of Otolaryngology-Head and Neck Surgery, First Affiliated Hospital of Guangxi Medical University, China.

### Cell culture

Nine NPC cell lines (HONE1, TWO3, HK1, HK1-EBV, 5-8F, 6-10B, CNE1, CNE2, and CNE2-EBV) and one immortalized human nasopharyngeal epithelial cell line, NP69, were kindly provided by Professor Sai-Wah Tsao (The University of Hong Kong), and cultured as required [23]. HK1-EBV cell line was identified by STR DNA profiling analysis.

### Immunohistochemistry (IHC)

IHC was performed as previously described [24]. Staining intensity was scored from 0 to 3 as: 0 (negative), 1 (weak), 2 (moderate), and 3 (strong). Percentage staining was evaluated on a scale from 0 to 4 as: 0 (less than 5%), 1 (5–25%), 2 (26–50%), 3 (51–75%), and 4 (76–100%). The final IHC score (0–12) was calculated by multiplying the intensity score by the percentage score.

### Reverse transcription-quantitative polymerase chain reaction (RT-qPCR)

Total RNA was extracted using the TRIzol reagent (Invitrogen, Carlsbad, CA, USA). cDNA was synthesized using a QuantiTect Reverse Transcription Kit (Cat. 205,313; Qiagen, Germany), using 500 ng of total RNA. One microliter of cDNA was used for real-time PCR using the QuantiTect SYBER Green PCR Master Mix assay (Applied Biosystems). The mRNA level of the target gene was calculated by the 2^−ΔΔCT^ method and normalized with GAPDH.

### siRNA transfection

Two different siRNAs of *TFRC* (si*TFRC*#1: SASI_Hs02_00330586, si*TFRC*#2: SASI_Hs02_00330588) and one siRNA of the universal negative control (SIC001) were designed and synthesized by the Sigma-Aldrich company for transient silencing in HK1-EBV cells. Transient transfection was performed using Lipofectamine 3000 (Invitrogen, Waltham, MA, United States) at a final siRNA concentration of 32 nM for 48 h. The transfected cells were harvested and used for subsequent experiments.

### FerroOrange

FerroOrange (#F374; Dojindo, Japan), a ferrous ion fluorescent probe, was used to detect intracellular ferrous ions. si*TFRC*#1-HK1-EBV, si*TFRC*#2-HK1-EBV, and siCtrl-HK1-EBV cells were seeded into an eight-chamber glass slide and incubated overnight. Cells were washed with HBSS three times and incubated with 1 µmol/L FerroOrange working solution for 30 min, protected from light. The cells were observed under a fluorescence microscope (Olympus, Tokyo, Japan).

### Cell proliferation assay

The effect of silencing the *TFRC* gene on cell growth was tested using the Cell Counting Kit-8 (CCK-8) (Dojindo, Kumamoto, Japan). In short, si*TFRC*#1-HK1-EBV, si*TFRC*#2-HK1-EBV, and siCtrl-HK1-EBV cells (2 × 10^3^) were seeded into 96-well plates. Subsequently, the cell proliferation capacity was measured every 24 h for 5 days at an OD of 450 nm using a microplate reader. The experiment was performed with five replicates.

Additionally, a colony formation assay was performed to determine the effect of *TFRC* silencing on colony formation. Transfected cells (800 cells) were seeded into six-well plates and cultured for 12 days. After washing with ice-cold PBS, the expanded colonies were fixed in 70% ethanol and stained with 0.1% crystal violet for 10 min. The plates were washed with water and dried at room temperature. The number of clones was then counted. The experiment was performed in triplicates under independent conditions.

### Cell cycle and cell apoptosis analysis using flow cytometry

The Muse Cell Cycle Kit (MCH100106; MUSE, Merck) and The Muse Annexin V and Cell Death Kit (MCH100105, MUSE, Merck) were used to determine whether TFRC silencing induced cell cycle arrest and cell death, respectively, following the manufacturer’s protocol. The samples were analyzed by a Muse cell analyzer (Millipore, USA). All experiments were performed in triplicate.

### Wound-healing assay

Transfected cells were planted in six-well plates. When cell density was about 90%, a sterile 200-µl pipette tip, guided by the sterile straightedge, was used to make four scratches across the plates. The medium was then changed to a low-FBS (1%) medium. Wound closure images were obtained at the same position using a light microscope at 0, 6, and 24 h. The wound healing results were calculated as the percentage of gap closure: (0h^Area^ − 6 h or 24h^Area^) / 0h^Area^ × 100%. All experiments were performed in triplicates.

### Transwell invasion assay

Transfected cells were harvested and resuspended in serum-free medium at a density of 3 × 10^5^ cells/ml, and then 500 µl of suspension mixture was added into the upper chamber of 24-well transwell chambers (8 μm) coated with Matrigel (Corning Incorporated, Corning, NY, United States). RPMI-1640 medium supplemented with 10% FBS was added to the lower chamber. After 72 h, non-invading cells were removed using cotton swabs, and invading cells were fixed, stained, and counted by averaging four fields under a light microscope.

### RNA-seq

RNA-seq was performed using si*TFRC*#2-HK1-EBV and siCtrl-HK1-EBV cells. All samples were prepared in triplicates. Total RNA was extracted using the NucleoSpin RNA Plus Kit (740984.10, Duren, Germany), following the manufacturer’s protocol. The sample quality was assessed using a Nanodrop spectrophotometer and agarose gel electrophoresis. Samples were sequenced using Takara. Sample library preparation was performed using the SMART-Seq kit, and sequencing was performed on the Novaseq6000 platform. The raw data were converted to Transcripts Per Million (TPM) file format. To avoid the influence of genes with a TPM value of zero, all values were converted to log (TPM + 0.001, 2) values. Differentially expressed genes (DEGs) were analyzed using independent-sample t-tests. DEGs were defined as a p-value < 0.05, and an absolute value of fold change > 2. Finally, these genes were entered into the Database for Annotation, Visualization, and Integrated Discovery (DAVID) database (https://david.ncifcrf.gov/) for gene ontology (GO) and Kyoto Encyclopedia of Genes and Genomes (KEGG) pathways analyses.

### In vivo xenograft experiments

Four-week-old male athymic BALB/c nude mice were purchased (n = 10 mice per group) from Japan SLC Inc. (Hamamatsu, Japan) and housed under specific pathogen-free conditions in accordance with the National Institute of Health guidelines. Cells transfected with si*TFRC*#2-HK1-EBV were used for nude mouse experiments because they showed better knockdown than si*TFRC*#1-HK1-EBV. Each mouse was subcutaneously injected with 2 × 10^6^ HK1-EBV cells (in 100 µl PBS) transfected with si*TFRC*#2 or siCtrl into the left flank. One mouse from the si*TFRC*#2-HK1-EBV group died accidentally on day 12. Body weight was monitored every 2 days, and tumor growth was monitored every 2 days once it became palpable. The tumor volume was calculated using the following formula: volume = (length × width^2^) / 2. The tumor cells were allowed to grow for 19 days, after which the mice were sacrificed, and the tumor tissues were weighed and collected for further staining.

### Western blotting

Extract total protein and perform western blotting as previous described [25]. The antibodies used in this study are summarized in Additional file 1. GAPDH was used as a loading control. Bands were quantified using ImageJ software. All targets were evaluated in triplicates. Uncropped western blotting membranes were showed in Additional file 2.

### Statistical analysis

The unpaired Student’s t-test was used to compare statistical differences between two groups, and a one-way analysis of variance was used to compare statistical differences between more than two groups. p < 0.05 were considered significant. The results are expressed as mean ± standard deviation (SD). All data were analyzed using the GraphPad Prism 9.0 program.

## Results

### TfR was significantly up-regulated in NPC

**Fig. 1:**
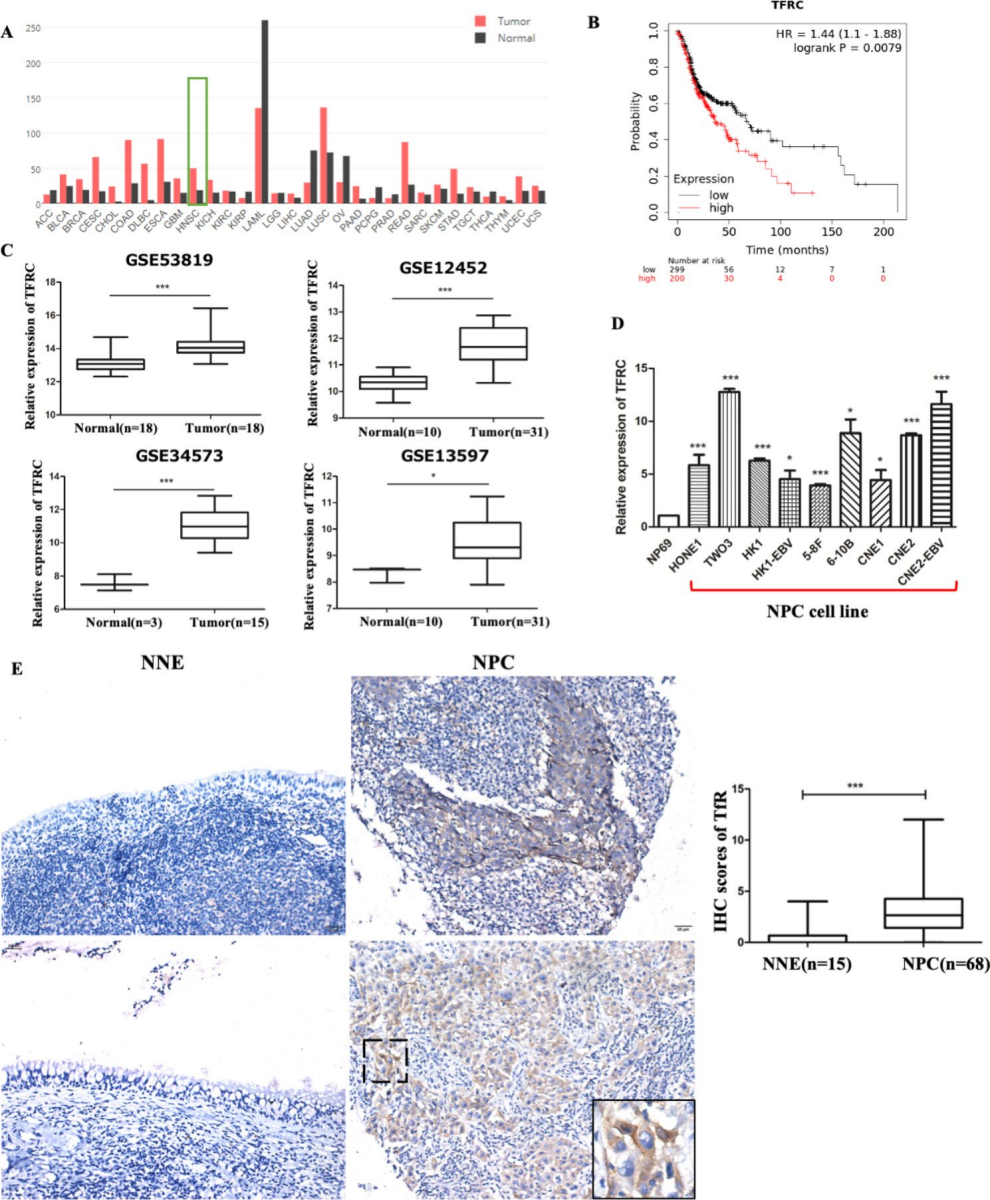
TfR was upregulated in cancers, including nasopharyngeal carcinoma (NPC). (**A**) GEPIA database showed *TFRC* was widely upregulated in most cancer types, including head and neck squamous carcinoma (HNSC). (**B**) High *TFRC* expression in HNSC patients is associated with shorter overall survival. (**C**) *TFRC* was upregulated in NPC tissues than control tissues in four NPC GEO datasets. (**D**) *TFRC* was upregulated in 9 NPC cell lines than normal nasopharyngeal epithelial cell line NP69, as assessed by RT-qPCR. (**E**) TfR was upregulated in NPC tissues by IHC. Asterisks indicate *p < 0.05, ***p < 0.001 compared to control

To explore the role of *TFRC* in NPC development, public datasets were used to study the levels of *TFRC* in cancer and normal individuals. By analyzing TCGA data using the GEPIA database, we found that *TFRC* was upregulated in most types of cancer (22/31) (Fig. 1A), including head and neck squamous carcinoma (HNSC) (Fig. 1A, green-framed) (full name of abbreviations are in Additional file 3). Survival analysis using the HNSC’s Meier plotter database revealed that patients with high expression of *TFRC* had poorer overall survival (Fig. 1B). Consistent upregulation of *TFRC* was found in NPC tissues compared to that in normal tissues in four GEO datasets (GSE53819, GSE12452, GSE34573, and GSE13597) (Fig. 1C).

To validate the upregulation of *TFRC* in NPC, we confirmed its upregulation in our samples by RT-qPCR and IHC analysis. *TFRC* mRNA levels were significantly upregulated in nine NPC cell lines compared with those in the normal nasopharyngeal epithelial cell line, NP69, as determined by RT-qPCR (Fig. 1D). Moreover, we investigated TfR protein expression in NPC tissue microarrays and non-cancerous epithelial tissues using IHC. Although two tissues in the NPC microarray were destroyed during the IHC procedure, we found that the expression of TfR was significantly upregulated in NPC tissues (n = 68) compared with that in control epithelial tissues (n = 15). As shown in Fig. 1E (enlarged in inset), TfR was expressed in both the cytoplasm and membrane. The expression of TfR in the tumor cells of the cancer nest was higher than that in the surrounding non-tumor cells. However, non-cancerous epithelial tissues exhibited weak expression. Taken together, our data indicate that TfR is overexpressed at the mRNA and protein levels in NPC cells.

### TFRC knockdown inhibited the proliferation of NPC cells

**Fig. 2:**
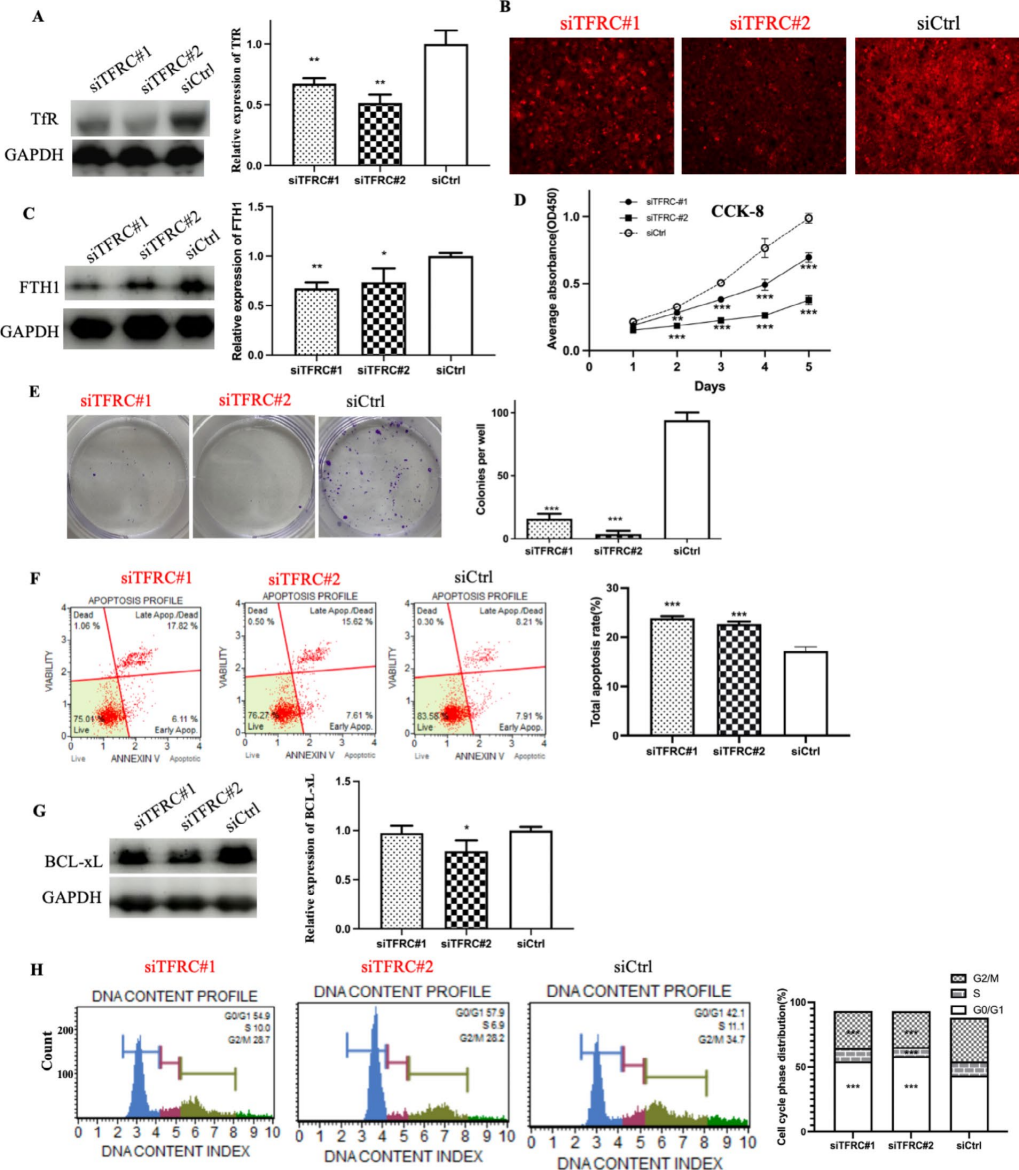
*TFRC* knockdown inhibited cell growth in vitro. (**A**) Successful knockdown of TFRC levels using two different siRNAs in HK1-EBV cells. (**B**) The intracellular Fe^2+^ visualized using FerroOrange was decreased after *TFRC* knockdown compared to the level in control cells. (**C**) Knockdown of *TFRC* inhibited the iron storage protein FTH1. (**D**) The CCK-8 assays showed knockdown of *TFRC* significantly suppressed cell proliferation from day 2 compared to that in control cells. (**E**) Representative images from colony formation cells, and the quantitative data indicate that knockdown of *TFRC* inhibited the colony formation ability in NPC cell line. (**F**) Representative distribution of the apoptotic cells using the Muse cell analyzer, and the quantitative data indicating total apoptotic cells significantly increased in si*TFRC*#1(23.9%) and si*TFRC*#2(22.7%) than that in the control (17.1%). (**G**) Knockdown of *TFRC* inhibited anti-apoptosis protein BCL-xL. (**H**) Representative cell cycle plots for the cells using Muse™ Cell analyzer, and the quantitative data indicating a significant arrest of si*TFRC*#1 group (54.4%) and si*TFRC*#2(58.4%) cells in the G0/G1 cell cycle phases compared to that in the control (43.5%). The results are given for three independent experiments. Asterisks indicate *p < 0.05, **p < 0.01, ***p < 0.001 compared to control

Given the abnormally high expression of TfR in NPC cells, TfR may play an important role in maintaining malignant biological behavior, and knockdown of *TFRC* may inhibit NPC progression. HK1-EBV cell line was chosen for further experiments because this cell line is still EBV positive and green fluorescence (EBV) could be detected in our lab. We established *TFRC* transient silencing in HK1-EBV cells by transfecting the cells with two siRNAs. Western blot analysis confirmed that the protein level of TfR was significantly reduced by 33% in si*TFRC*#1-HK1-EBV cells and by 49% in si*TFRC*#2-HK1-EBV cells compared with that in siCtrl-HK1-EBV cells (Fig. 2A).

The effect of *TFRC* knockdown on intracellular iron content was investigated. The amount of intracellular iron was detected using FerroOrange, and the protein level of FTH1, which encodes the major intracellular iron storage protein, was detected using western blotting. Intracellular iron levels decreased after *TFRC* knockdown compared with those in control cells (Fig. 2B). Western blot confirmed that the protein level of FTH1 was significantly decreased after *TFRC* knockdown compared with that in control cells (Fig. 2C). These results indicate that *TFRC* knockdown leads to iron deprivation in NPC cells.

To determine the effect of *TFRC* on tumor cell growth, CCK-8 and colony formation assays were conducted. As shown in Fig. 2D, the growth of *TFRC* knockdown si*TFRC*#1-HK1-EBV and si*TFRC*#2-HK1-EBV cells was significantly attenuated compared with that in siCtrl cells. The colony formation assay also revealed that knockdown of *TFRC* remarkably suppressed clone formation abilities compared to that in the siCtrl group (Fig. 2E).

### TFRC knockdown triggered apoptosis and cell cycle arrest

Flow cytometry was used to measure the effects of *TFRC* knockdown on cell death and cell cycle progression. As shown in Fig. 2F, total apoptotic rates were significantly higher in si*TFRC*#1 (23.9%) and si*TFRC*#2 (22.7%) than in the control (17.1%). The increase in the apoptotic rate was mainly due to late apoptosis. Western blot analysis demonstrated that the knockdown of *TFRC* in si*TFRC*#2 significantly inhibited the anti-apoptotic protein BCL-xL compared to that in siCtrl cells (Fig. 2G), leading to more apoptotic cells. Furthermore (Fig. 2H), we observed a significant cell cycle arrest in the G0/G1 phase (si*TFRC*#1: 54.4% and si*TFRC*#2: 58.4%) compared with that in the control (43.5%). In addition, the rate of S phase and G2/M phase (S phase of si*TFRC*#1: 10%, si*TFRC*#2: 7%, G2/M phase of si*TFRC*#1: 29%, si*TFRC*#2: 28%) was decreased in *TFRC* knockdown cells compared with that in control cells (S phase: 11%; G2/M phase: 34%), although no significant difference was observed in the S phase of si*TFRC*#1-HK1-EBV cells. These results suggest that *TFRC* knockdown triggers cell cycle arrest and apoptosis in NPC cells.

### TFRC knockdown suppressed cancer cell migration, invasion, and EMT

**Fig. 3:**
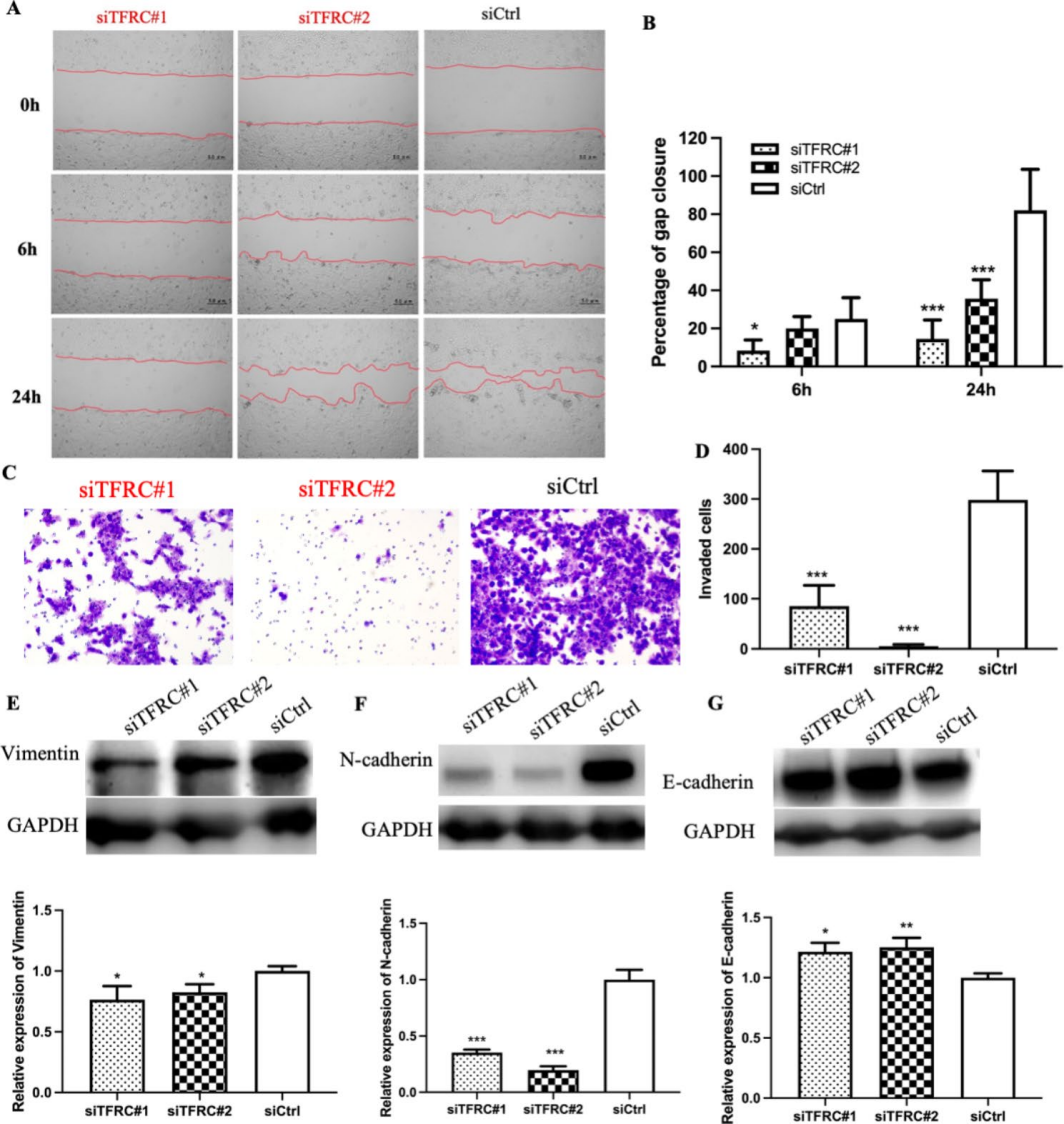
Knockdown of *TFRC* inhibited cell migration, invasion, and epithelial–mesenchymal transition in vitro. (**A**) Representative images from wound healing assays showing knockdown of *TFRC* suppressed cell migration. (**B**) Summary bar graph showing percentage wound closure in *TFRC* knockdown group is lower than that in the control group at the 6 and 24 h time points. (**C**) Representative images of the invasion assay. (**D**) Summary bar graph showing the invaded cells in the *TFRC* knockdown cells as less than that in the control cells. (**E-G**) Knockdown of *TFRC* inhibits Vimentin and N-cadherin (mesenchymal phenotype), and increases E-cadherin (epithelial phenotype). The results are given for three independent experiments. Asterisks indicate *p < 0.05, **p < 0.01, ***p < 0.001 compared to control

Cell migration and invasion are important hallmarks of cancer and remain the main contributors to cancer-associated deaths. Migration was assessed using a wound healing assay (Fig. 3A, B). Six or twenty-four hours after the scratch, the rates of migration in si*TFRC*#1-HK1-EBV cells (6 h: 8.3%; 24 h: 14.5%) and si*TFRC*#2-HK1-EBV cells (6 h: 20.1%; 24 h: 35.7%) were significantly lower than those in control cells (6 h: 25.1%; 24 h: 82.0%). Furthermore, a transwell assay with Matrigel was used to determine the invasion ability of the cells (Fig. 3C, D). The number of invaded cells was also significantly decreased in both si*TFRC*#1-HK1-EBV cells (86) and si*TFRC*#2-HK1-EBV cells [6], compared with that in the control groups (298).

These results suggested that *TFRC* knockdown suppressed the migration and invasion of NPC cells. Since EMT is required for tumor invasion and metastasis, EMT-associated proteins were assessed using western blotting. The results showed that, compared with control cells, mesenchymal phenotype proteins (Vimentin and N-cadherin, Fig. 3E, F) were significantly decreased, while epithelial phenotype protein (E-cadherin, Fig. 3G) was significantly increased in *TFRC* knockdown cells. These findings suggest that *TFRC* knockdown inhibits cell migration and invasion by suppressing EMT in NPC.

### Knockdown of TFRC suppressed tumor formation in nude mice

**Fig. 4:**
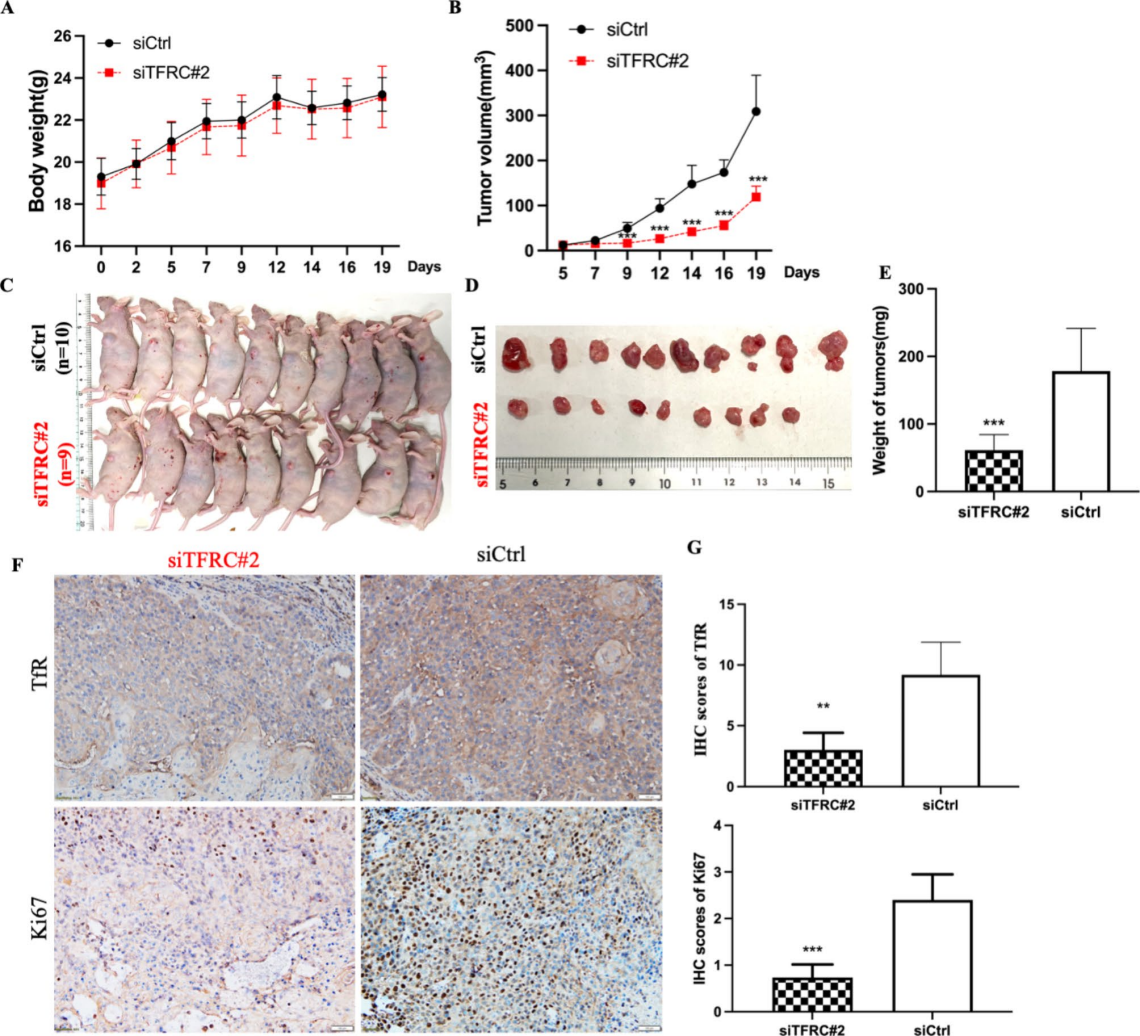
Xenograft tumor formation of HK1-EBV cell with knockdown of *TFRC* in nude mice. (**A**) There was no difference on mouse body weight. (**B**) Growth curves showed that knockdown of *TFRC* significantly decreased tumor volume from day 9 compared that in control mouse. (**C**) Nude mice were sacrificed 19 days after initial implantation and (**D**) surgically excised tumor tissues. (**E**) Average weight of implanted tumors in si*TFRC*#2-HK1-EBV and siCtrl-HK1-EBV xenograft-bearing mice. (**F, G**) The xenograft tumors were stained and show that TfR level was significantly downregulated, and the Ki67 staining was significantly decreased in *TFRC* knockdown group compared with control group, as assessed by IHC. Asterisks indicate **p < 0.01, ***p < 0.001 compared to control

Then, we investigated whether *TFRC* knockdown represses tumor formation in vivo. After the subcutaneous injection of si*TFRC*#2-HK1-EBV and siCtrl-HK1-EBV for 19 days, the mice were sacrificed. There was no significant difference in the body weight between the two groups during tumor growth (Fig. 4A). The tumor volume in the si*TFRC*#2-HK1-EBV mice were significantly lower than those in control mice after day 9 (Fig. 4B). Tumors derived from the si*TFRC*#2-HK1-EBV group were smaller (Fig. 4 C, D), and the mean tumor weight was also lighter in si*TFRC*#2-HK1-EBV group (61.3 mg) compared with those in the siCtrl-HK1-EBV group (178.4 mg, Fig. 4E). IHC analysis of mouse tumor samples showed that the expressions of *TFRC* and Ki-67 were downregulated in the tumor tissues of mice with knockdown of *TFRC* (Fig. 4F, G). These findings suggest that knockdown of *TFRC* might suppress tumor growth in vivo.

### Identification of TFRC downstream effectors in NPC cells

**Fig. 5:**
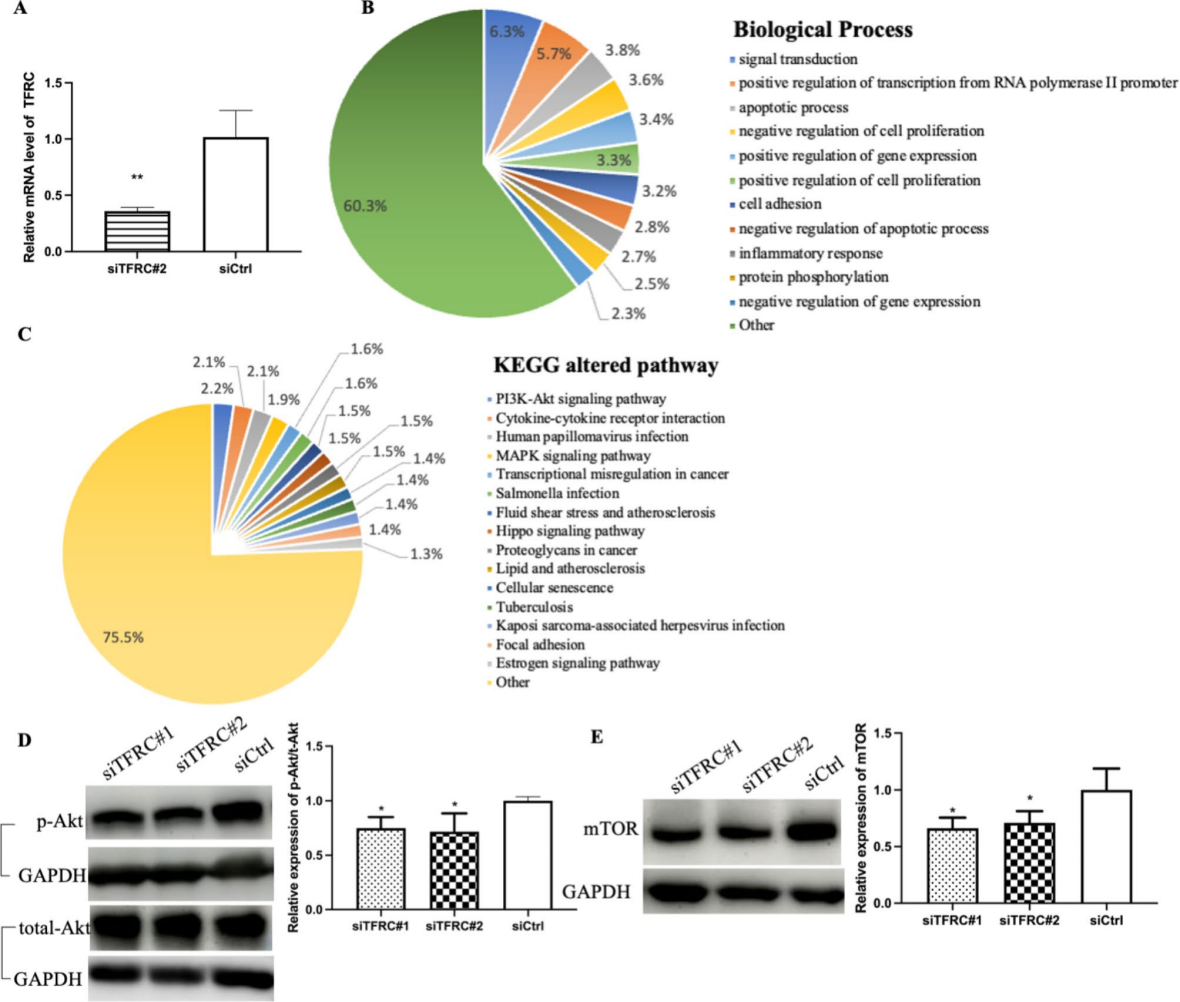
RNA sequencing analysis after *TFRC* knockdown in HK1-EBV cells. (**A**) mRNA level of *TFRC*  was significantly decreased in si*TFRC*#2-HK1-EBV samples, as assessed by RT-qPCR. (**B**) Proportion pie chart showing GO analysis of altered biological process after knockdown of *TFRC*, using DAVID database. (**C**) Proportion pie chart showed altering pathways after knockdown of *TFRC*, using KEGG data from DAVID database. (**D**) Knockdown of *TFRC* inhibited the phospho-Akt (p-Akt) level, which is the PI3K-Akt signaling pathway marker. (**E**) Knockdown of *TFRC* inhibited the mTOR level. The results are given for three independent experiments. Asterisks indicate *p < 0.05 compared to control

To further explore the mechanism by which *TFRC* knockdown inhibits NPC, RNA-seq was used to perform a DEGs analysis of si*TFRC*#2-HK1-EBV and siCtrl-HK1-EBV. As shown in Fig. 5A, the samples were confirmed by RT-qPCR and showed a significant decrease of *TFRC*. RNA-seq showed that among the DEGs in NPC cells, 385 genes were upregulated and 432 genes were downregulated. To clarify the possible influence of *TFRC* knockdown, the DEGs were analyzed using DAVID database. In biological process terms (Fig. 5B), the DEGs in HK1-EBV cells with *TFRC* knockdown were mainly enriched in signal transduction, regulation of transcription from the RNA polymerase II promoter, apoptotic processes, cell proliferation, gene expression, cell adhesion, inflammatory response, and protein phosphorylation. Since KEGG pathway (Fig. 5C) indicated that the PI3K/Akt pathway was the top-ranked related pathway in our sequencing results and was closely related to the occurrence and development of tumors, we hypothesized that the PI3K/Akt pathway is the downstream mechanism of inhibiting NPC progression after *TFRC* knockdown. We then measured the protein levels of the PI3K/Akt pathway-related protein phospho-Akt (p-Akt) and its downstream molecule, mTOR, using western blotting. Our validation data showed that the protein levels of p-Akt (Fig. 5D) and mTOR (Fig. 5E) were remarkably reduced in *TFRC*-knockdown cells compared with those in control cells. These results indicated that *TFRC* knockdown may inhibit cancer progression via the PI3K/Akt/mTOR signaling pathway.

## Discussion

Our previous study showed that iron deprivation decreases the proliferation of NPC cells. TfR is a cellular iron gate that plays a critical role in maintaining cellular iron homeostasis. This study showed that *TFRC* was overexpressed in NPC tissues. Moreover, we found that *TFRC* knockdown led to iron deprivation and inhibited proliferation, migration, invasion, and EMT via the PI3K/Akt/mTOR signaling pathway. To our knowledge, this is the first study to assess TfR expression and the effects of *TFRC* knockdown on NPC progression.

Tumors require sufficient iron input to drive their proliferation and progression. The iron input receptor TfR is overexpressed in a variety of solid tumor types and is associated with a worse prognosis. However, carcinoid, prostate, and testicular cancers show low expression [26]. Cui et al. found that *TFRC* downregulation promotes cancer progression [27]. Thus, the role of *TFRC* in cancer progression remains unclear. In our study, we found a similar expression in most tumors, and *TFRC* overexpression was also observed in NPC. In addition, *TFRC* overexpression can be associated with poor prognosis and is considered an effective prognostic marker in different types of cancer, such as esophageal squamous cell carcinoma [21], breast cancer [28, 29], renal cell carcinoma [30], and hepatocellular carcinoma [31]. Interestingly, patients with non-Hodgkin’s lymphoma (NHL) who are positive for human immunodeficiency virus (HIV) present with a higher level of *TFRC* than do patients with NHL who are HIV-negative. Another study found that targeting anti-TfR antibodies is a promising approach to prevent Epstein–Barr Virus (EBV) carcinogenesis [32]. TfR appears to be associated with viral infections. It is well known that elevated plasma EBV is related to NPC [33]. Further research is required to clarify the relationship between TfR and EBV infection in NPC.

Elevated TfR levels and its core role in cancer pathology provide new insights into cancer therapies. Currently, there are two methods of TfR-targeted cancer therapy. Indirect methods use TfR antibodies conjugated with anticancer drugs such as chemotherapeutics, toxins, and nucleic acids. Direct methods include direct silencing of *TFRC* or activation of antibody-mediated effector functions such as antibody-dependent cell-mediated cytotoxicity (ADCC), antibody-dependent cell-mediated phagocytosis (ADCP), and complement-dependent cytotoxicity (CDC) [34–37]. Direct methods limit iron uptake, impair TfR function, and lead to iron deprivation. Direct anticancer activity of TfR has been confirmed in many types of cancer [38–40]. Our research also found *TFRC* knockdown by siRNA could significantly inhibit the growth, migration, and invasion of NPC cells, which is consistent with previous studies on most cancers [18, 41, 42]. These findings indicate that *TFRC* knockdown may be a promising therapeutic target for NPC.

The mechanism by which TfR affects cancer progression varies among cancer types. Huang et al. found that TfR promotes proliferation and metastasis by upregulating AXIN2 expression in epithelial ovarian cancer cells [18]. Jeong et al. reported that TfR promotes the growth of human pancreatic ductal adenocarcinoma by increasing ROS and mitochondrial respiration [43]. O’Donnell et al. confirmed that TfR is a downstream target of the c-Myc oncogene in B-cell lymphoma [44]. To clarify the possible mechanism by which TfR affects NPC progression, we analyzed the GO and KEGG pathways of TfR using the DEGs after RNA-sequencing. GO analysis showed that proliferation, apoptosis, and cell adhesion may be related to *TFRC* knockdown. These findings were consistent with our experimental results. By sequencing, we identified a series of cancer-related pathways, including the PI3K/Akt signaling pathway, cytokine–cytokine receptor interaction, human papillomavirus infection, and the MAPK signaling pathway. KEGG pathway analysis showed that the PI3K/Akt signaling pathway was the most affected pathway after *TFRC* knockdown. Activation of the PI3K/AKT signaling pathway promotes mTOR activation. Therefore, mTOR expression was investigated using western blotting. We found that *TFRC* knockdown inhibited cancer progression via the PI3K/Akt/mTOR signaling pathway. The PI3K/AKT/mTOR pathway is one of the most frequently altered pathways in cancer. Previous studies have shown that the PI3K/AKT/mTOR pathway modulates cell cycle, apoptosis, autophagy, angiogenesis, EMT, and chemoresistance in cancers [45, 46]. Additionally, drugs targeting the PI3K/AKT/mTOR pathway in combination with chemotherapeutic drugs are considered promising treatment approaches [47]. Several genes have been reported to influence cell cycle, apoptosis, migration, and invasion via the PI3K/AKT/mTOR signaling pathway in cancer [48–50]. Moreover, Zhang et al. [51] reported that proliferation of human mesangial cells and PI3K/Akt/mTOR activation were dependent on TfR, by showing that PI3K/Akt/mTOR was inhibited by the human TfR polyclonal antibody. These evidences suggested that TFRC might regulate the PI3K/Akt-mTOR pathway to affect the development of NPC. Therefore, we hypothesized that *TFRC* knockdown inhibits NPC progression by inhibiting the PI3K/AKT/mTOR pathway. We confirmed that p-Akt and mTOR, which are important markers of the PI3K/AKT/mTOR pathway, were decreased in *TFRC*-knockdown cells compared with the levels in control cells. These results indicated that *TFRC* knockdown may inhibit cancer progression via the PI3K/Akt/mTOR signaling pathway.

Our study still has several limitations that need to be noted. As we show in Additional file 4, using the publicly available GEO dataset (GSE102349), which contains 73 NPC patients, we demonstrate that there is a positive correlation between TRFC expression level and clinical stage. In order to determine whether it can be employed as a prognostic marker for NPC, additional studies using samples from the NPC cohort with detailed clinical information are required. It is also important to confirm that the further overexpression of TFRC could promote NPC progression.

## Conclusions

**Fig. 6:**
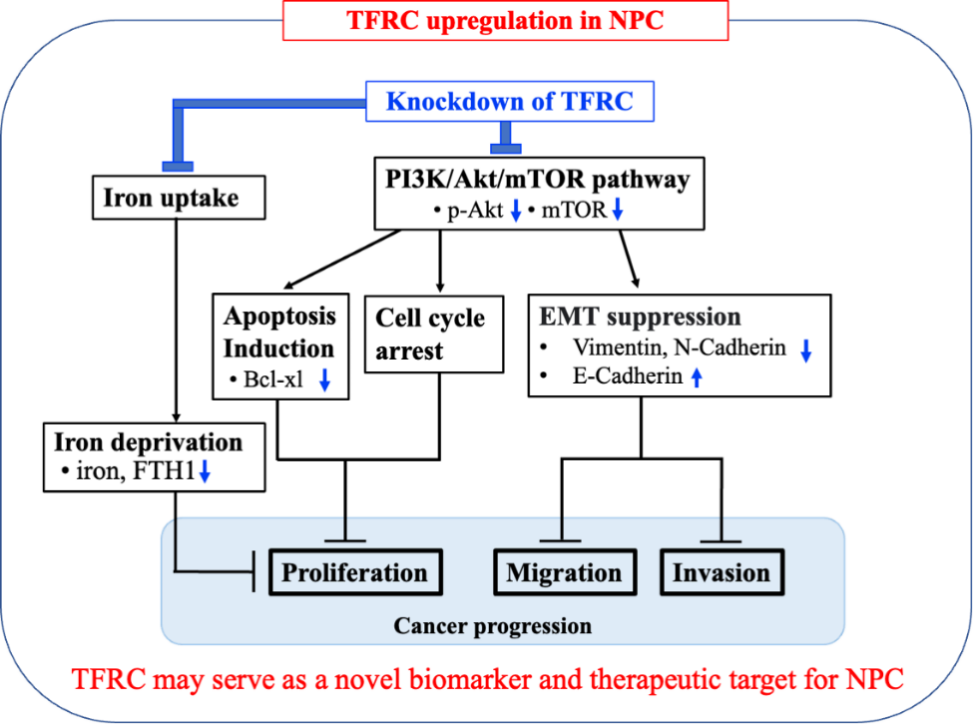
Schematic diagram of the proposed mechanisms. TfR was overexpressed in NPC tissues and cells. Knockdown of *TFRC* led to iron deprivation and inhibited cell proliferation, migration, invasion, and EMT, which may function by inhibiting the activity of the PI3K/Akt/mTOR signaling pathway. *TFRC* participates in NPC development and may serve as a novel biomarker and therapeutic target for NPC.

In summary, in this study, we found that TfR was overexpressed in NPC tissues and cells. Knockdown of *TFRC* led to iron deprivation and inhibit cell proliferation, migration, invasion, and EMT, which may function by inhibiting the activity of the PI3K/Akt/mTOR signaling pathway. These results indicated that *TFRC* participates in NPC development and may serve as a novel biomarker and therapeutic target for NPC (Fig. 6).

### Electronic supplementary material

Below is the link to the electronic supplementary material.

**Additional file 1:** Showed the antibodies used in this study.

**Additional file 2:** Showed the uncropped western blotting membranes.

**Additional file 3:** Showed the full list of abbreviations.

**Additional file 4:** Showed the correlation between *TFRC* expression level and clinical stage in GSE102349.

## Data Availability

The data and materials in the current study are available from the corresponding author on reasonable request.
