# Supplementary figures and images for: Knockdown of TFRC suppressed the progression of nasopharyngeal carcinoma by downregulating the PI3K/Akt/mTOR pathway
Source: Cancer Cell Int. 2023 Aug 29;23:185. doi: 10.1186/s12935-023-02995-7 (PMC10466839; doi:10.1186/s12935-023-02995-7)

Figure 2A

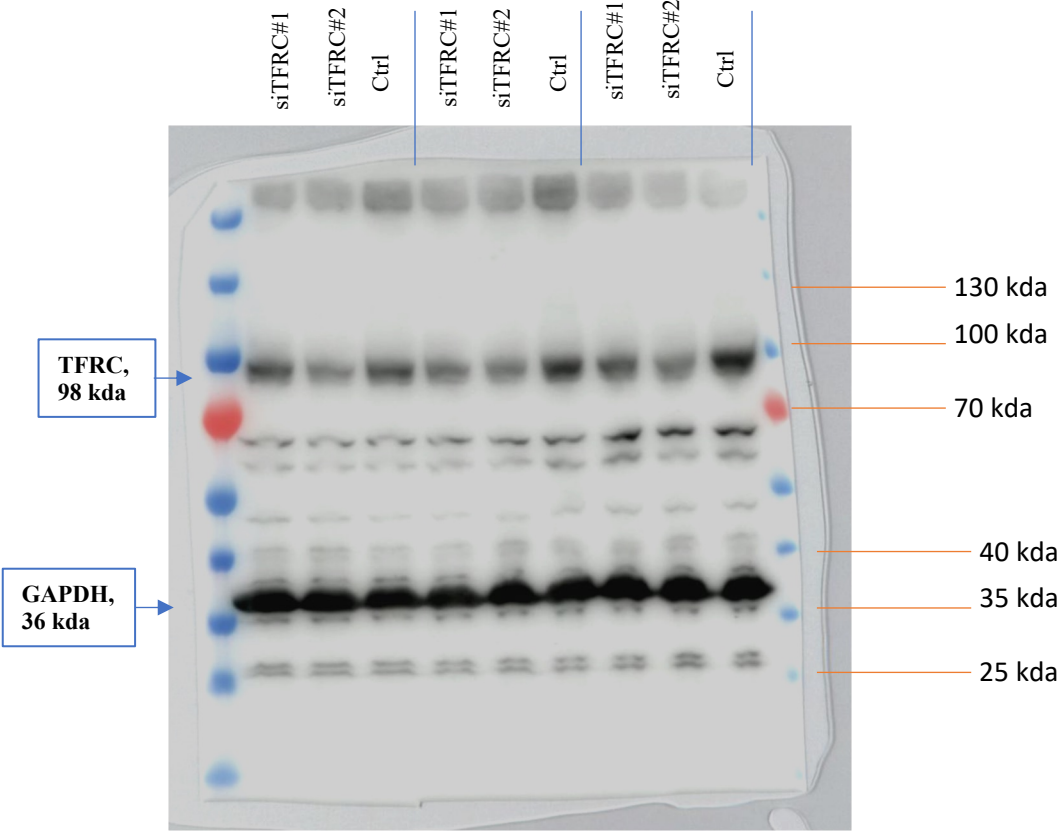

Figure C

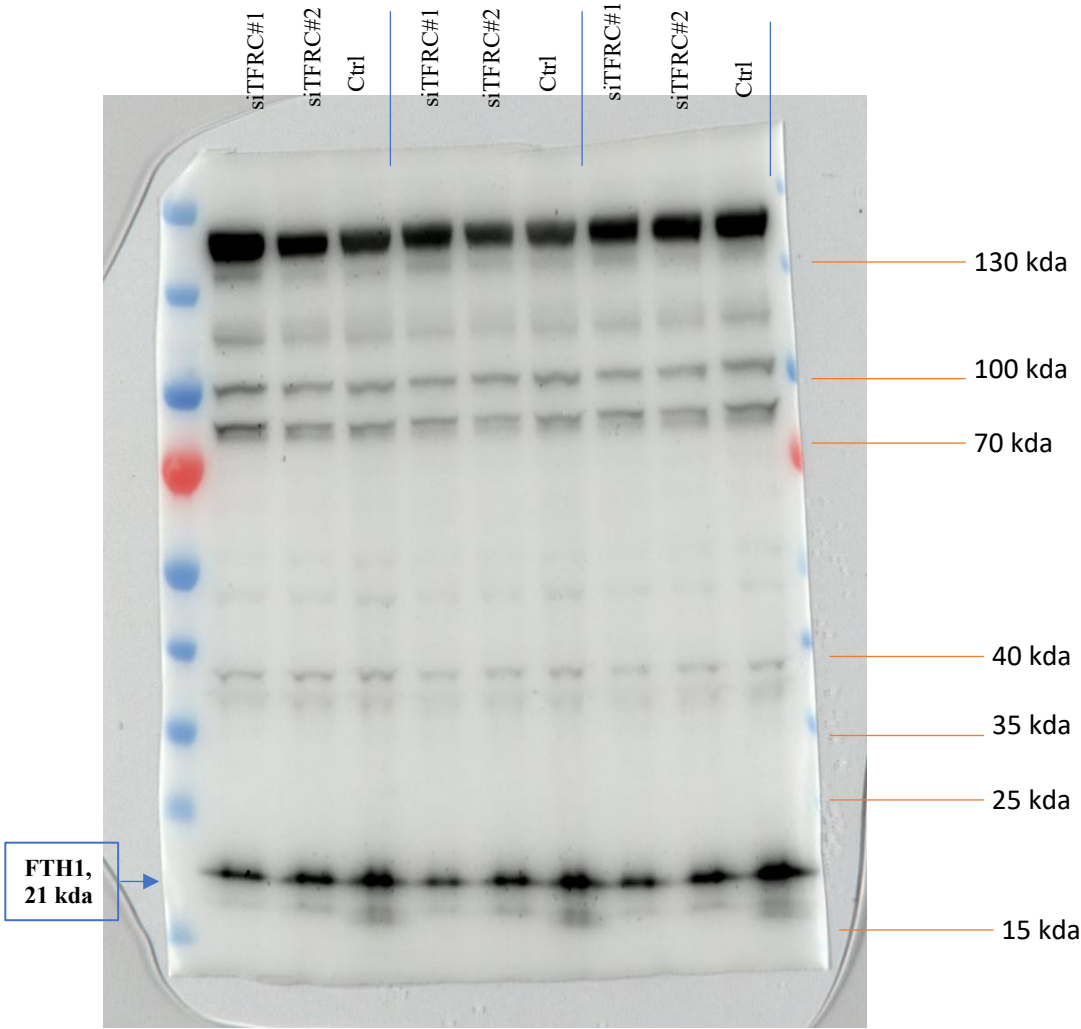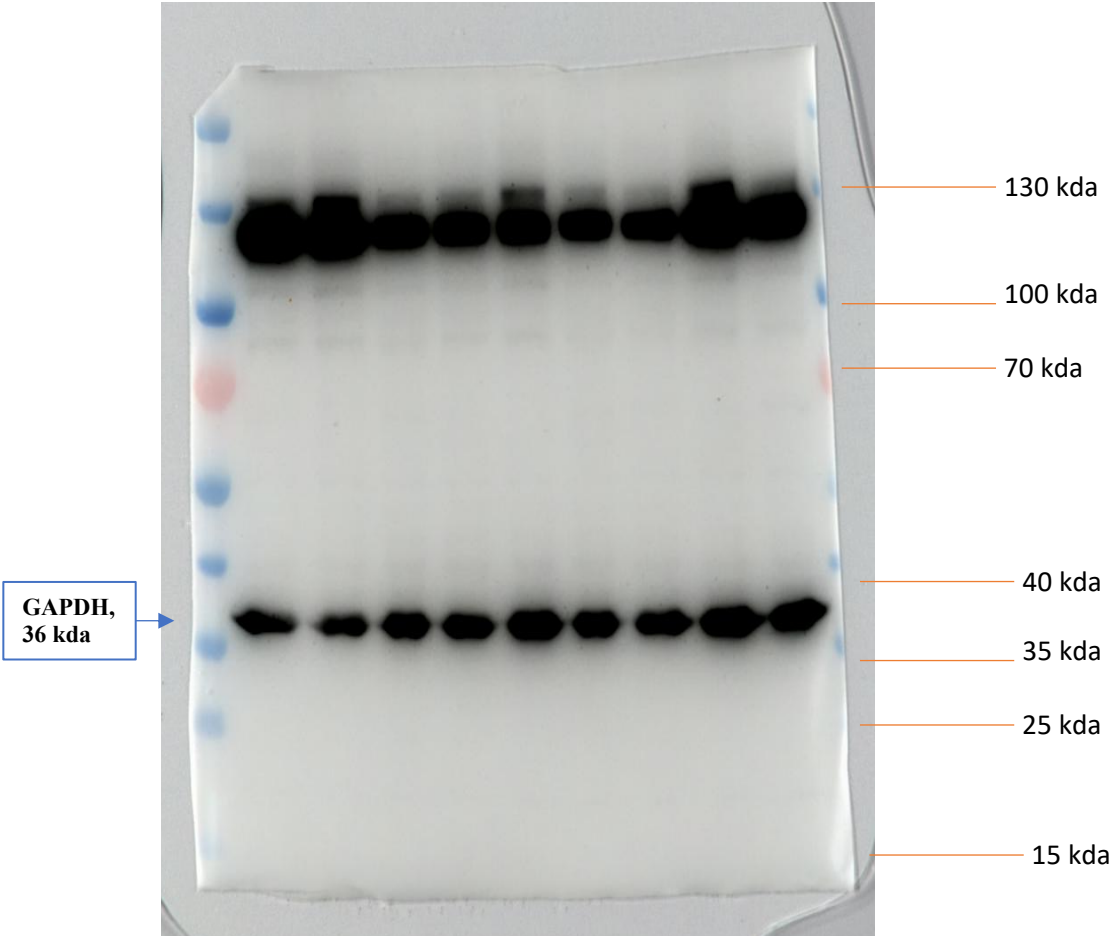

Figure 2H

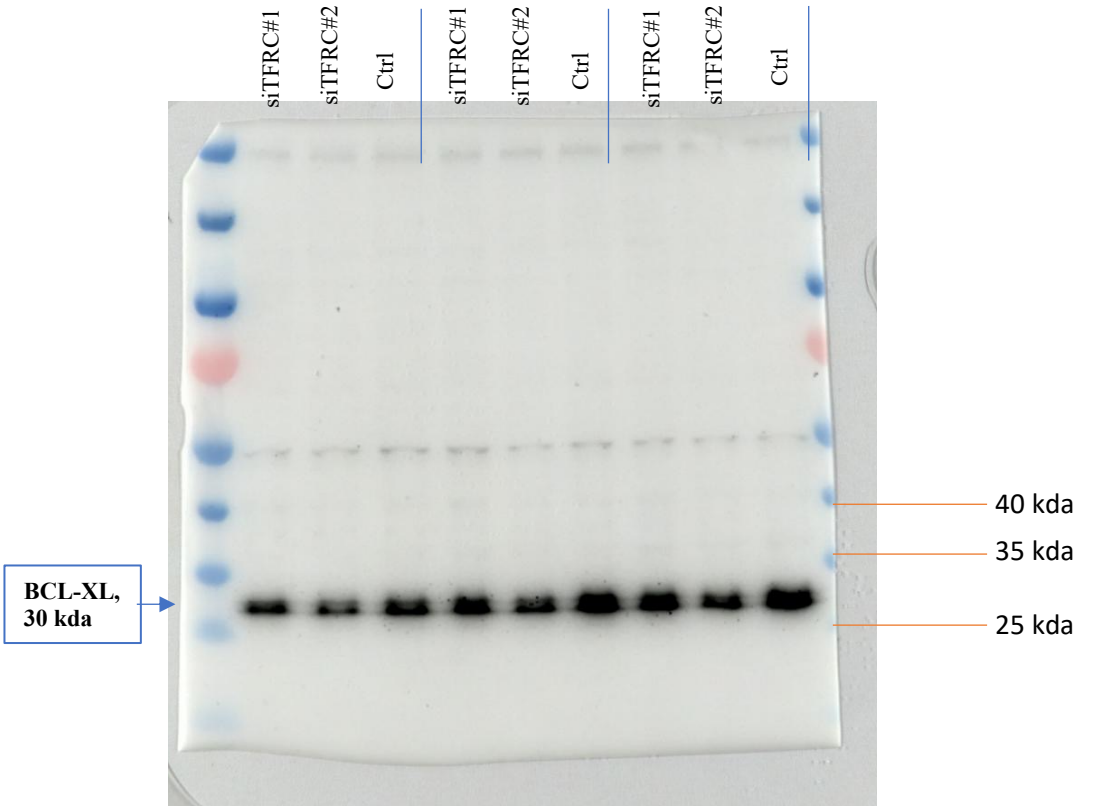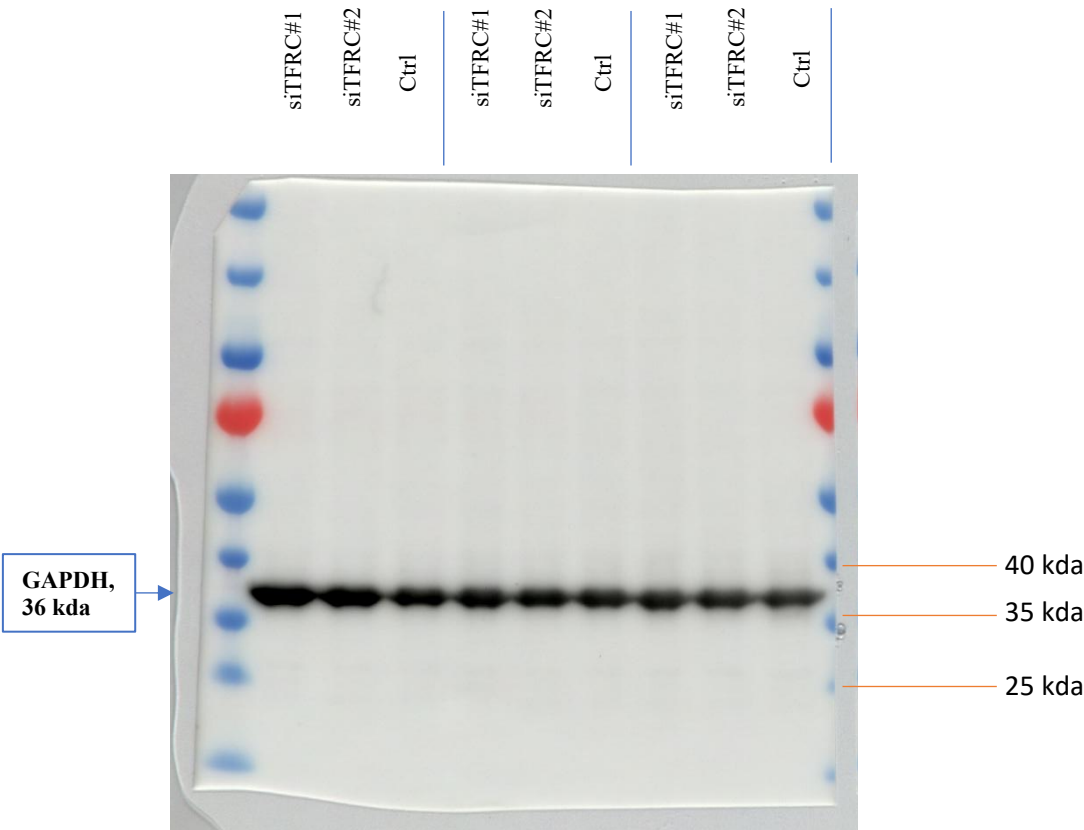

Figure 3E

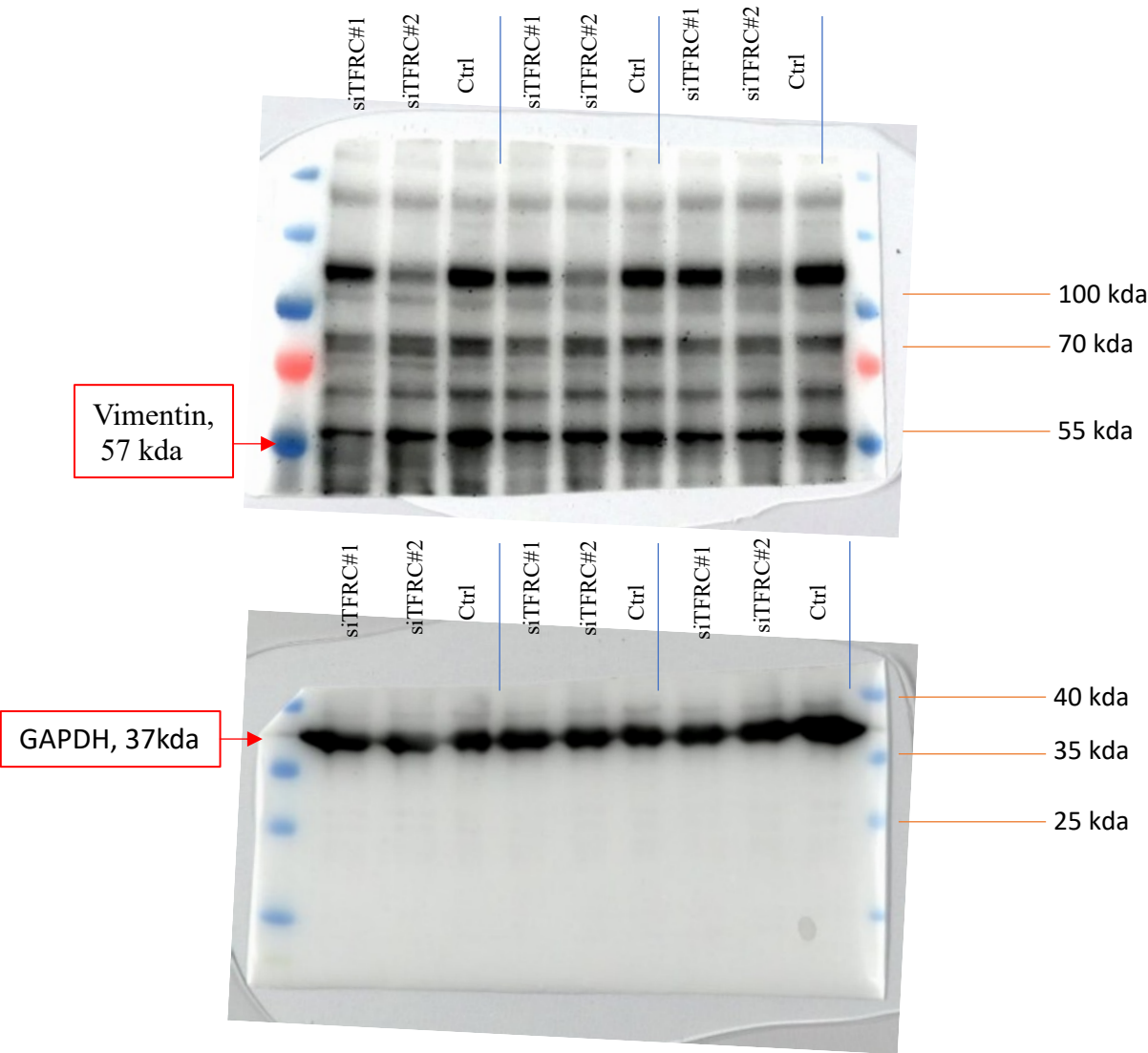

### Figure 3F

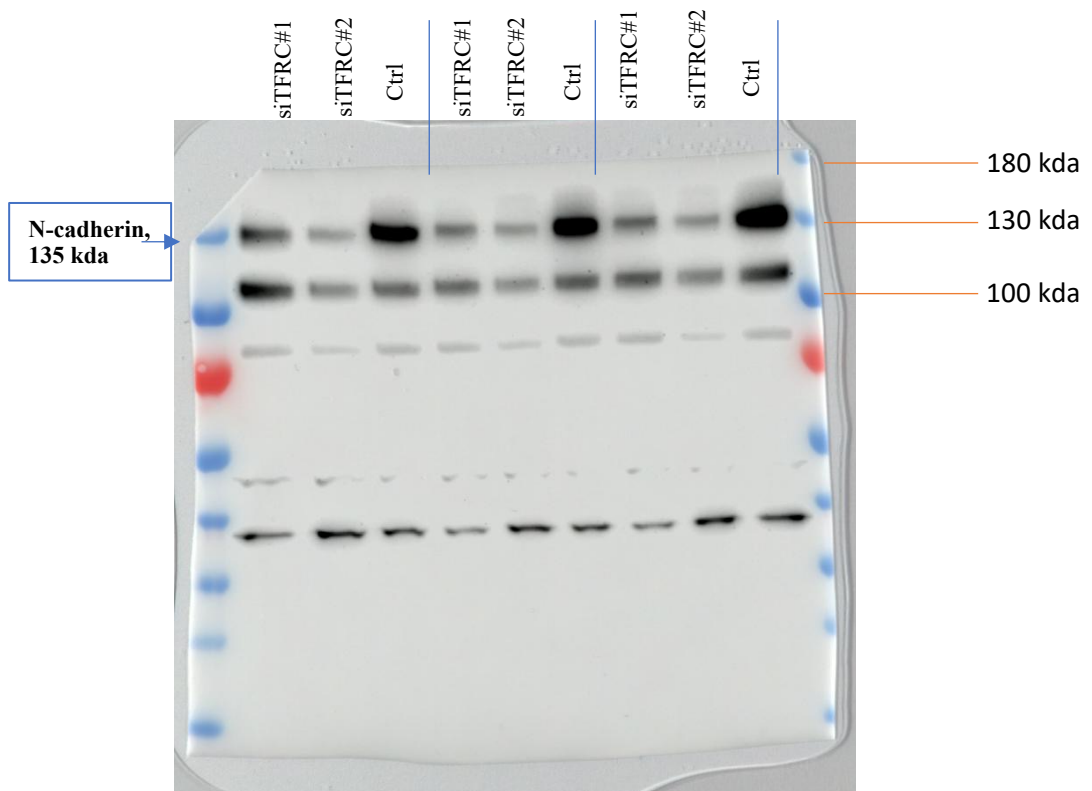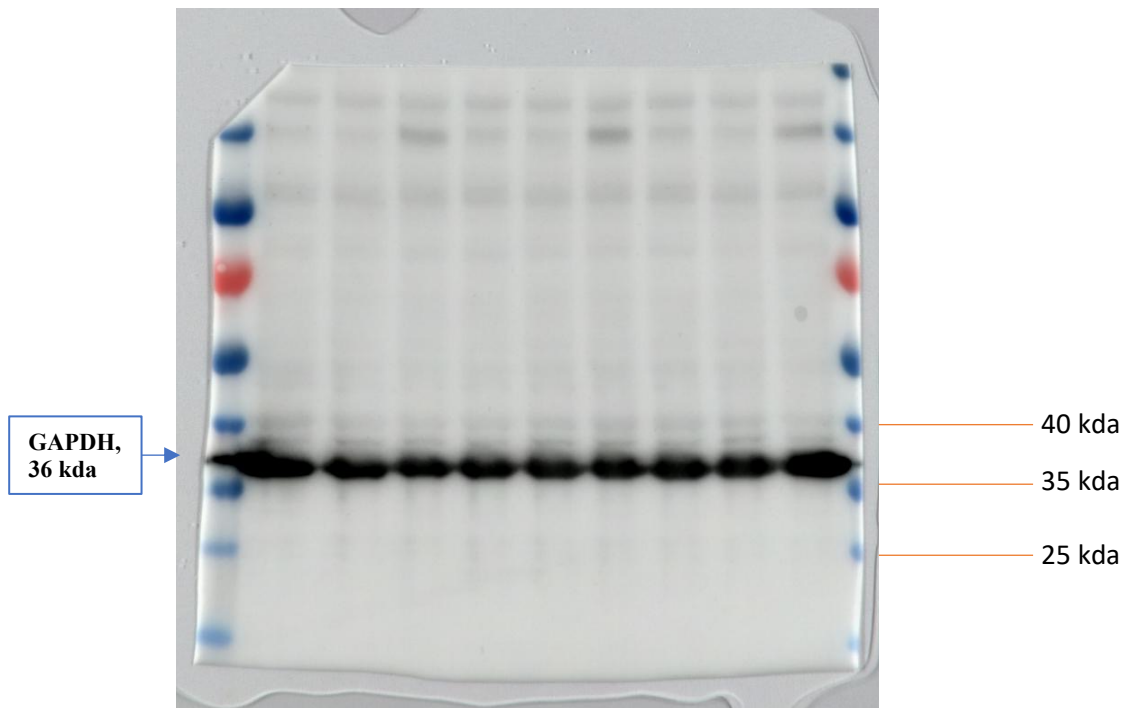

Figure 3G

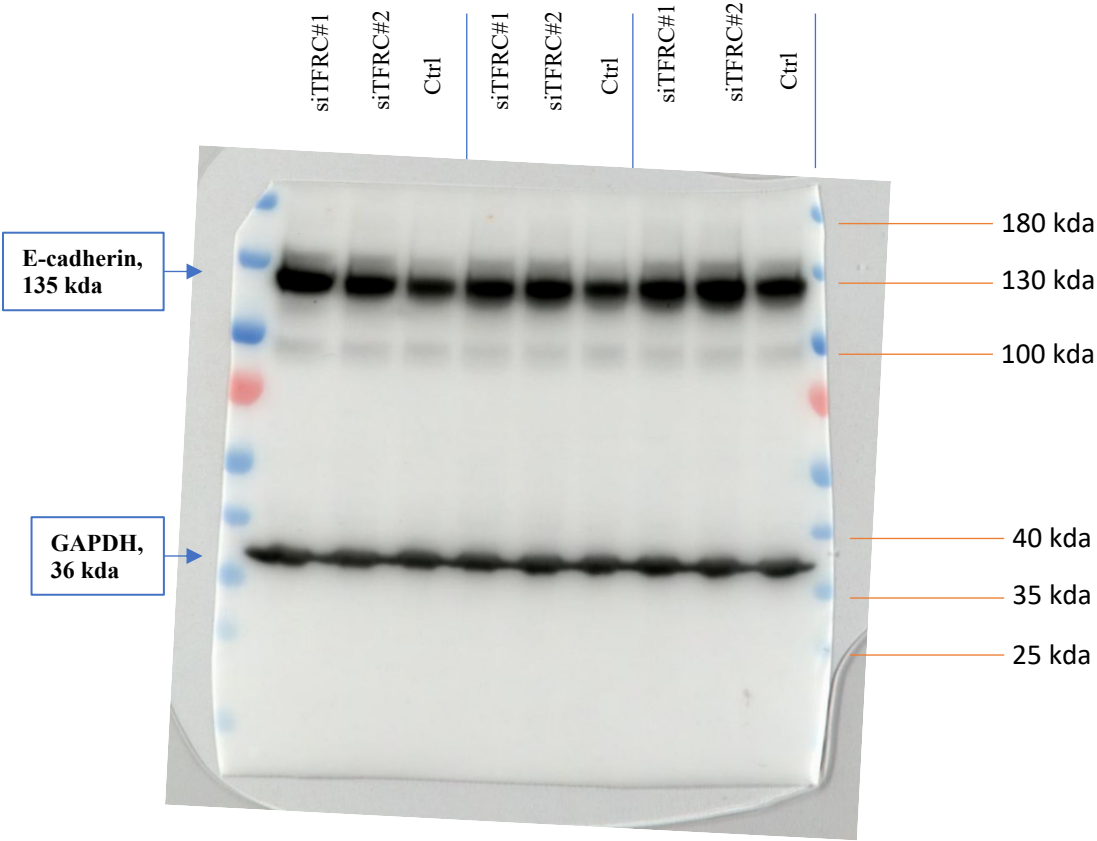

Figure 5D

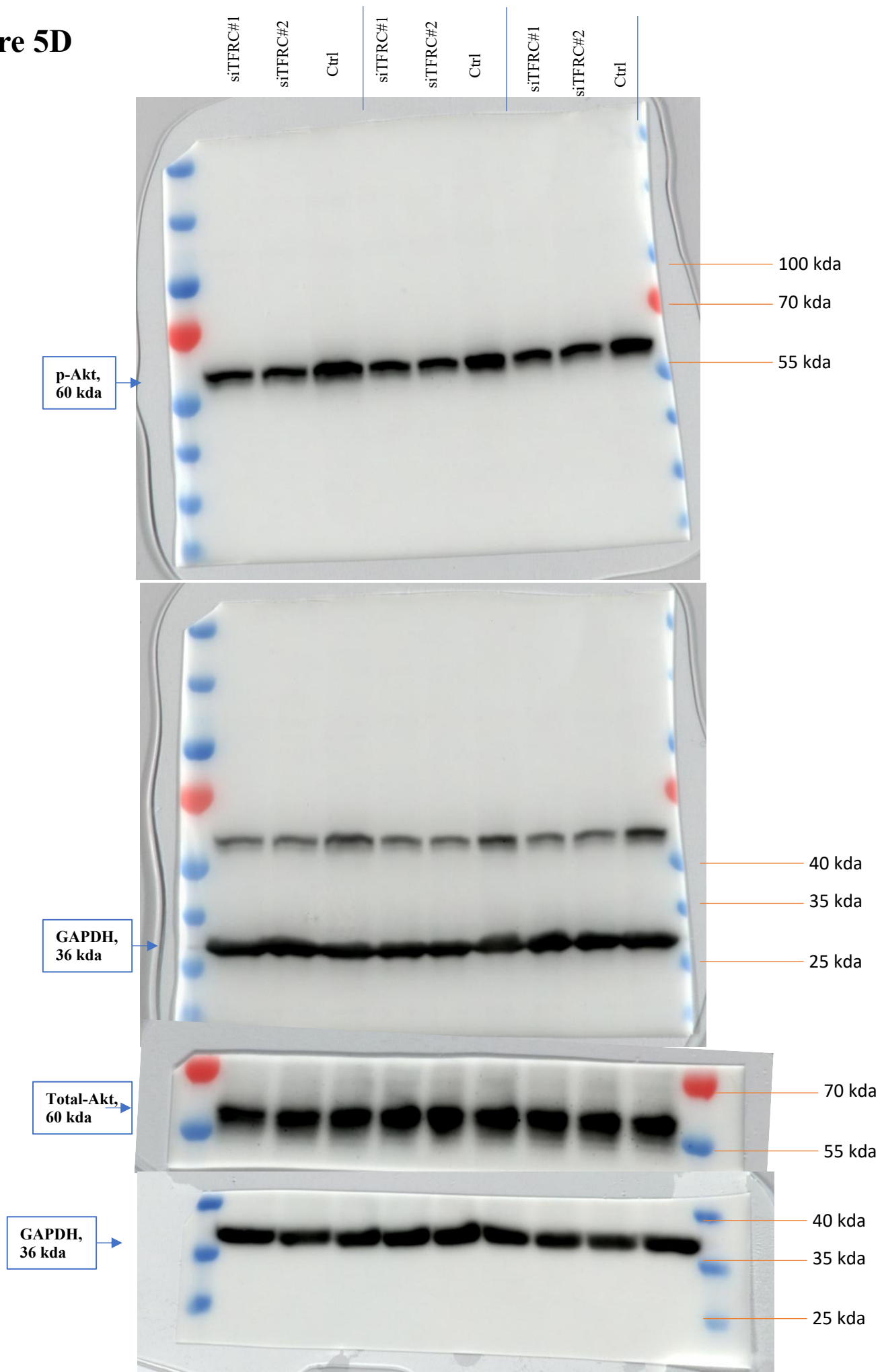

Figure 5E

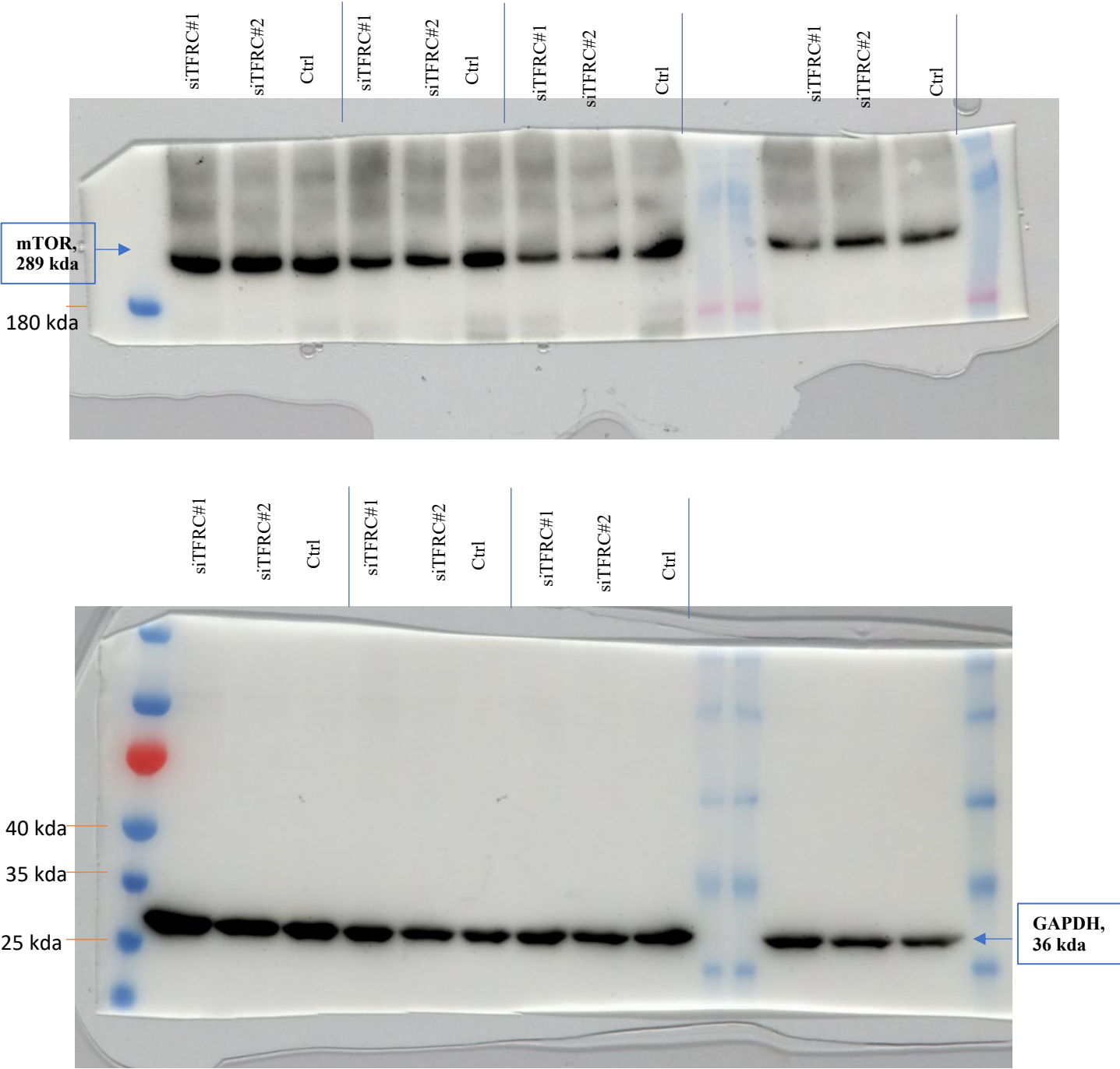

Supplement: Supplementary file 2 — Additional file 2: Showed the uncropped western blotting membranes. [file 12935_2023_2995_MOESM2_ESM.pdf]

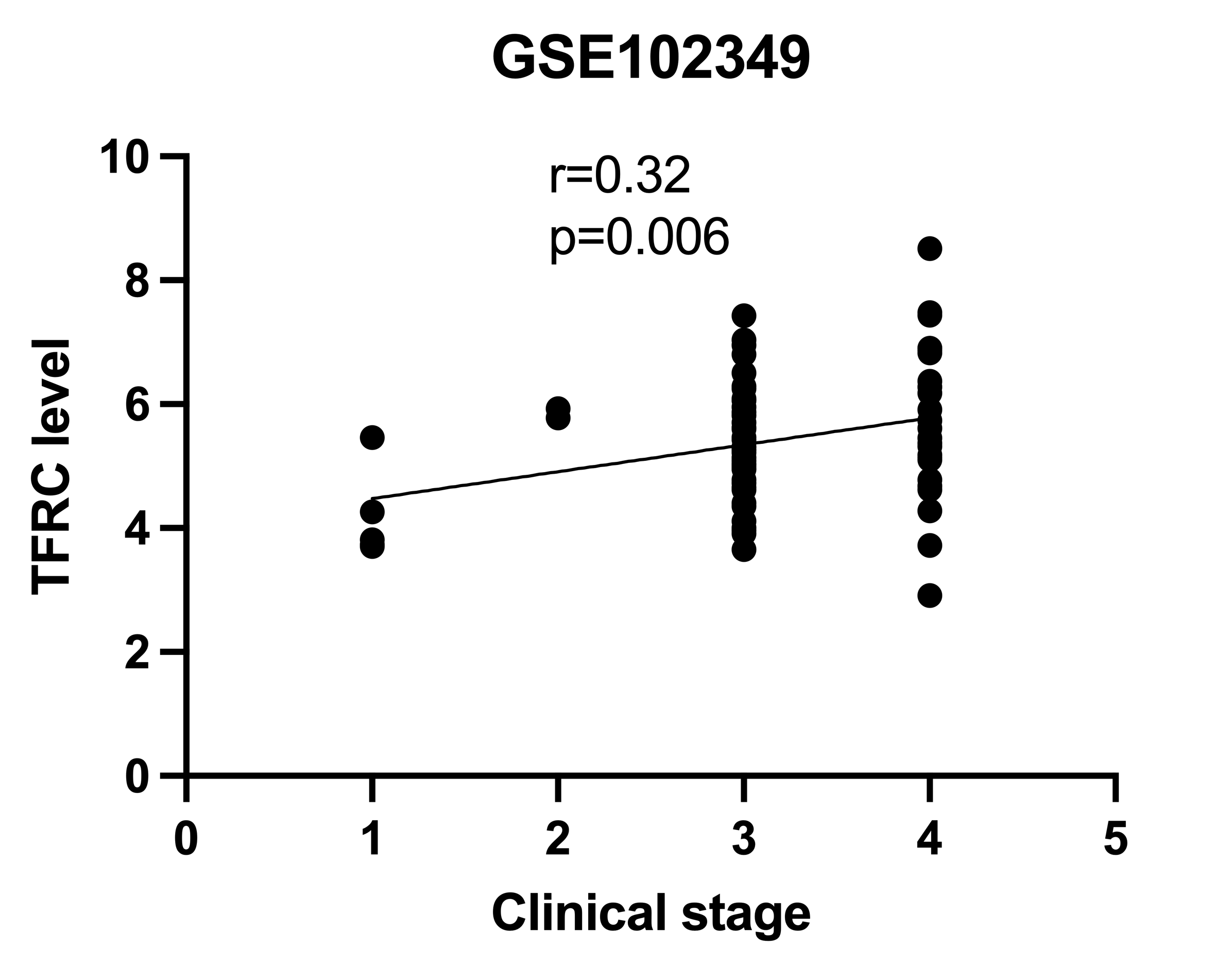

Supplement: Supplementary file 4 — Additional file 4: Showed the correlation between TFRC expression level and clinical stage in GSE102349. [file 12935_2023_2995_MOESM4_ESM.png]
